# Recombinant Cpn 0810 stimulates proinflammatory cytokine expression and apoptosis in human monocytes

**DOI:** 10.3892/etm.2014.2111

**Published:** 2014-12-05

**Authors:** YUYU CHEN, BAIPING WU, LIANGZHUAN LIU, XIAOXING YOU, LILI CHEN, YIMOU WU, QIUGUI ZHANG

**Affiliations:** 1 Department of Inspection, The Affiliated Cancer Hospital of Xiangya School of Medicine, Central South University, Changsha, Hunan 410013, P.R. China; 2 Department of Pathogenic Biology, University of South China, Hengyang, Hunan 421001, P.R. China; 3 Department of Inspection, The First Affiliated Hospital of University of South China, Hengyang, Hunan 421001, P.R. China

**Keywords:** Cpn 0810, *Chlamydophila pneumoniae*, THP-1 cells, proinflammatory cytokines, apoptosis

## Abstract

The aim of the present study was to express the recombinant *Chlamydophila pneumoniae* (*C. pneumoniae*) protein, Cpn 0810, in *Escherichia coli* (*E. coli*) BL21, and investigate the effects of Cpn 0810 on inflammatory and apoptotic processes in human monocytic (THP-1) cells. An ELISA was performed to detect the levels of the proinflammatory cytokines, tumor necrosis factor (TNF)-α and interleukin (IL)-6. In addition, Hoechst 33258 staining and annexin V binding analyses were performed to measure the rates of apoptosis. Purified glutathione S-transferase (GST)-Cpn 0810 recombinant proteins were obtained from the *E. coli* BL21 cells carrying the pGEX6p-2/Cpn 0810 plasmid, and were shown to stimulate the expression of TNF-α and IL-6 in the THP-1 cells in a dose- and time-dependent manner. TNF-α and IL-6 levels peaked at 24 h after GST-Cpn 0810 stimulation. Furthermore, GST-Cpn 0810 significantly promoted the apoptosis of THP-1 cells. In conclusion, recombinant GST-Cpn 0810 was shown to stimulate the expression of TNF-α and IL-6, inhibit proliferation and induce apoptosis in THP-1 cells. Therefore, Cpn 0810 may interact with host cells following *C. pneumoniae* infection, functioning as an important pathogenic factor.

## Introduction

*Chlamydophila pneumoniae* (*C. pneumoniae*) is a common respiratory pathogen that can cause a number of respiratory diseases, including pneumonia, asthma, chronic pharyngitis and chronic bronchitis (1–4). *C. pneumoniae* activates macrophages to produce proinflammatory cytokines, which may result in atherosclerosis. Although *C. pneumoniae* is a serious threat to human health, its underlying pathogenic mechanisms are not fully understood. It has, however, been hypothesized that *C. pneumoniae* secretes various toxic proteins.

It is widely accepted that gram-negative bacteria secrete proteins through type I-V secretion systems. The type III secretion system (T3SS) is an independent system, whose effector proteins can change cytoskeletal structures, destroy signal transduction pathways, suppress apoptotic activity and interfere with host transcriptional regulation (5–7). Techniques for the screening and identification of Cpn T3SS have become increasingly studied. Previous studies have shown that the coding sequences of T3SS effector proteins are always located next to the chaperones (8–15). The Cpn 0810 gene is adjacent to Cpn lcrH1, a chaperone homolog gene with *Yersinia* lcrH, and the Cpn 0810 gene family is located within the coding clusters of the T3SS. Therefore, Cpn 0810 has been hypothesized to be an effector of the T3SS (16–19).

In the present study, Cpn 0810 was cloned, expressed and purified from *C. pneumoniae*. The effects of Cpn 0810 on inflammatory and apoptotic processes in human monocytic cells (THP-1) were investigated, with the aim to provide a basis for the further study of the pathogenic mechanisms underlying Cpn T3SS effector proteins.

## Materials and methods

### Strains, plasmids and cell lines

An *Escherichia coli* (*E. coli)* BL21 strain and the THP-1 cell line were provided by the Department of Pathogenic Biology, University of South China (Hengyang, China).

### Gene amplification and recombinant plasmid construction

Amplification of Cpn 0810 was performed using polymerase chain reaction (PCR), based on the following primer pairs: P1, 5′-CGCGGATCCATGAATAAAAAGCCCAAGAAAAC-3′, and P2, 5′-TTTTCCTTTTGCGGCCGCTTACTCAGC GCCTTTAACCAT-3′.

Amplification was performed in a final reaction volume of 50 μl, containing 39.6 μl ddH_2_O, 5 μl 10X Pfu buffer, 1 μl dNTP mix (10mM), 1 μl P1 primer, 1 μl P2 primer, 0.4 μl DNA Polymerase (5 units) and 2 μl Cpn templates. The amplification conditions were as follows: Initial polymerase activation at 94°C (5 min); 30 cycles of 94°C (30 sec), 52°C (45 sec) and 72°C (3 min); and a final elongation step at 72°C for 10 min. Distilled water was used as a negative control. The amplification products (363 bp) were subjected to 1.0% agarose gel electrophoresis containing ethidium bromide.

The PCR products were digested with *Bam*HI and *Not*I (Promega Corporation, Madison, WI, USA), and ligated into the pGEX6p-2 plasmid (GE Healthcare, Piscataway, NJ, USA). The recombinant plasmid was transformed into *E. coli* BL21 competent cells, and the positive clones were screened by PCR and sequencing.

### Expression and purification of the recombinant protein

Positive *E. coli* BL21 colonies, containing pGEX6p-2/Cpn 0810, were cultured in Luria-Bertani (LB) solid medium (with ampicillin) at 37°C overnight, after which the culture was transferred to fresh LB liquid medium (with ampicillin). When the optical density reached a wavelength of 600 nm, isopropyl β-D-1-thiogalactopranoside (IPTG) was added with a final concentration of 0.2 mM, and the culture was shaken at 30°C for 4 h. The bacteria were then collected, and phosphate-buffered saline (8 ml/g cells) and lysozyme (4.0 g/l) were added to the cell pellet. Following incubation at room temperature for 2 h, the cells were subjected to sonication (10 sec on, 10 sec off) 30 times using a MSE Soniprep 150 (SANYO, Osaka, Japan). Following centrifugation at 10,000 × g for 20 min at 4°C, the supernatant was purified using a glutathione S-transferase (GST) purification resin column (Novagen; Merck KGaA, Darmstadt, Germany), according to the manufacturer’s instructions. The GST-Cpn 0810 recombinant protein was identified by western blot analysis using a mouse anti-Cpn AR39 primary antibody (1:2,000 dilution; ab190064, Abcam, Cambridge, MA, USA), and the protein concentration was detected using bicinchoninic acid kits (Pik-day Bio Co., Ltd., Beijing, China).

### Cell culture and simulation

THP-1 cell lines were cultured in RPMI 1640 medium (GE Healthcare Life Sciences, Logan, UT, USA), supplemented with 10% fetal bovine serum (FBS; GE Healthcare Life Sciences) and 2 mmol/l glutamine, in a humidified incubator at 37°C with 5% CO_2_. For simulation, cells were seeded on plastic culture plates (Corning Inc., Corning, NY, USA) and cultured in 1% FBS overnight. Cells were then stimulated using specific concentrations of GST-Cpn 0810 for predetermined time periods.

### ELISA analysis

THP-1 cells were cultured in suspension, at a density of 10^6^ cells/ml, and seeded on 24-well plates. The groups were treated with 0.5, 1, 2, 3, 4, 5 and 6 μg/ml GST-Cpn 0810 in serum-free culture medium for 24 h. Treatments of 5 μg/ml GST and distilled water were used as negative controls, while 0.1 μg/ml lipopolysaccharide (LPS) treatment was used as a positive control. After 24 h, the supernatant was collected for analysis of tumor necrosis factor (TNF)-α and interleukin (IL)-6 by ELISA (Jingmei Biological Engineering Co., Ltd., Shenzhen, China). When the optimal concentration of GST-Cpn 0810 treatment was determined, the cells were cultured with the specific concentration of GST-Cpn 0810 for 0, 6, 12, 24, 36 and 48 h before the culture supernatant was used for TNF-α and IL-6 analysis.

### Hoechst 33258 staining

THP-1 cells were seeded on six-well plates, at a density of 5×10^5^ cells/ml, and stimulated with 0, 5 and 10 μg/ml GST-Cpn 0810 for 24 h. Apoptosis was subsequently analyzed using Hoechst staining kits (Beyotime Institute of Biotechnology, Haimen, China), according to the manufacturer’s instructions.

### Annexin V-fluorescein isothiocyanate-propidium iodide (FITC-PI) assay

THP-1 cells were seeded on six-well plates, at a density of 5×10^5^ cells/ml, and stimulated with 0, 5, 10, 15 and 20 μg/ml GST-Cpn 0810 for 18 h. The rates of apoptosis were analyzed using an annexin V-FITC apoptosis detection kit (KeyGen Biotech, Nanjing, China). Treatment with 5 μg/ml GST was used as negative control, 0.1 μg/ml LPS was used as a positive control and untreated cells were used as a blank control.

### Statistical analysis

Data are expressed as the mean ± standard deviation. Statistical analysis was performed using SPSS 13.0 software (SPSS, Inc., Chicago, IL, USA). Paired t-test was used to analyze comparisons between groups, and for analysis of paired data. P<0.05 was considered to indicate a statistically significant difference.

## Results

### Cloning, expression and purification of recombinant GST-Cpn 0810

**Figure 1:**
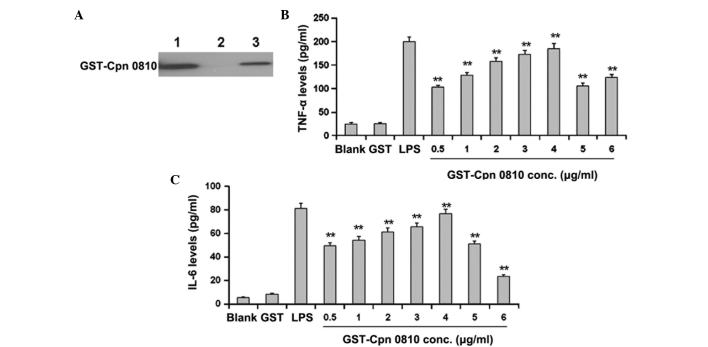
GST-Cpn 0810 increases the levels of proinflammatory cytokines in THP-1 cells in a dose-dependent manner. (A) Western blot analysis of the GST-Cpn 0810 recombinant proteins was performed with an anti-Cpn AR39 primary antibody. Lanes: 1, BL21 cells with pGEX6p-2/Cpn 0810 induced with 0.2 mmol/l IPTG; 2, non-induced BL21 cells; 3, purified GST-Cpn 0810 recombinant proteins. THP-1 cells were treated with GST-Cpn 0810 at gradient concentrations of 0.5, 1, 2, 3, 4, 5 and 6 μg/ml for 24 h, and the expression levels of (B) TNF-α and (C) IL-6 were analyzed with ELISA kits. ^**^P<0.01, vs. GST-treated group. GST, glutathione S-transferase; IPTG, isopropyl β-D-1-thiogalactopyranoside; TNF, tumor necrosis factor; IL, interleukin.

In order to obtain purified recombinant Cpn 0810, a pGEX6p-2/Cpn 0810 plasmid was constructed and transformed into *E. coli* BL21 cells. Following induction with 0.2 mM IPTG for 4 h, these cells were lysed and the supernatant was purified using a GST purification column. Western blot analysis was performed using anti-Cpn AR39 antibodies. A specific protein band was observed at approximately 42 kD, indicating the expression of recombinant GST-Cpn 0810 protein (Fig. 1A). This purified recombinant protein was used in the following experiments.

### Recombinant GST-Cpn 0810 elevates the expression levels of proinflammatory cytokines in THP-1 cells

To investigate the effects of GST-Cpn 0810 on the inflammation of THP-1 cells, purified recombinant GST-Cpn 0810 was incubated with the cells and the levels of TNF-α and IL-6 were detected using ELISA. THP-1 cells were treated with GST-Cpn 0810 at gradient concentrations of 0.5, 1, 2, 3, 4, 5 and 6 μg/ml for 24 h. The expression levels of TNF-α and IL-6 were found to increase over the concentration range, 0.5–4 μg/ml GST-Cpn 0810, when compared with the blank control group. Expression peaked at 4 μg/ml, where the expression levels of TNF-α and IL-6 were 184.75±17.40 and 75.36±29.49 pg/ml, respectively (Fig. 1B and 1C). At higher concentrations of 5–6 μg/ml GST-Cpn, the expression levels of TNF-α and IL-6 were reduced when compared with the peak levels. Accordingly, a concentration of 4 μg/ml GST-Cpn was used in the following time-course experiments.

**Figure 2:**
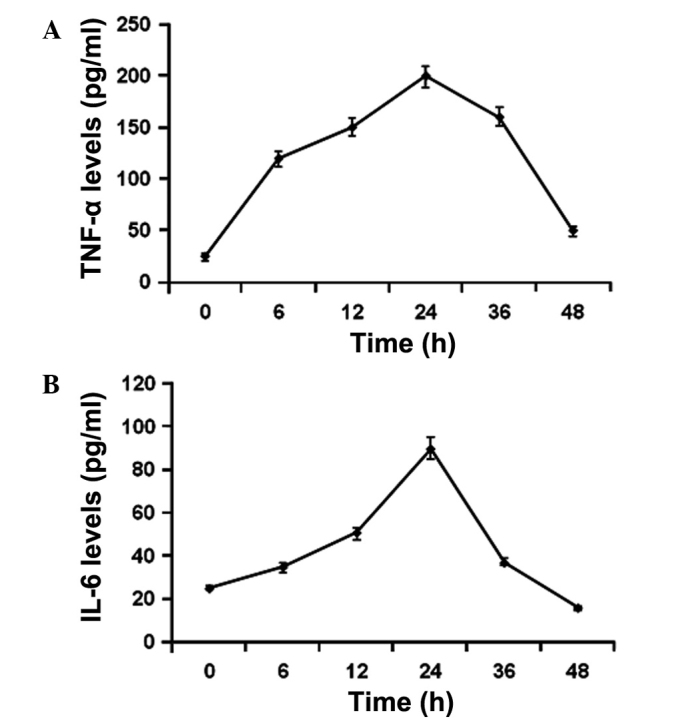
GST-Cpn 0810 increases the levels of proinflammatory cytokines in THP-1 cells in a time-dependent manner. THP-1 cells were treated with 4 μg/ml GST-Cpn 0810 for indicated time periods of 6, 12, 24, 36 and 48 h, and the supernatant was collected for (A) TNF-α and (B) IL-6 analysis. GST, glutathione S-transferase; TNF, tumor necrosis factor; IL, interleukin.

THP-1 cells were subsequently treated with 4 μg/ml GST-Cpn 0810 for 6, 12, 24, 36 and 48 h, and the supernatant was collected for the TNF-α and IL-6 assays. The results showed that the stimulated expression levels of TNF-α and IL-6 were detectable 6 h after GST-Cpn 0810 treatment, peaking at 24 h (Fig. 2). Therefore, the results indicated that GST-Cpn 0810 may elevate the levels of TNF-α and IL-6 expression in a dose- and time-dependent manner.

### Recombinant GST-Cpn 0810 promotes apoptosis in THP-1 cells

**Figure 3:**
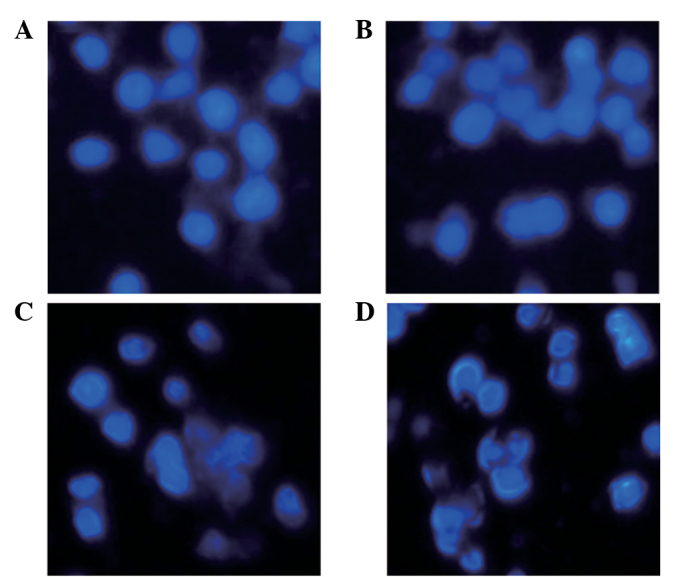
Hoechst 33258 staining of THP-1 cells in the (A) normal control, (B) GST-treated control, (C) 5 μg/ml GST-Cpn 0810 and (D) 10 μg/ml GST-Cpn 0810 groups (magnification, ×1,000). THP-1 cells were stimulated with 0, 5 and 10 μg/ml GST-Cpn 0810 for 24 h, and Hoechst 33258 staining was performed to analyze apoptosis. GST, glutathione S-transferase.

Hoechst 33258 staining and annexin V binding analyses were performed to investigate the impact of GST-Cpn 0810 on the apoptotic process of THP-1 cells. For Hoechst 33258 staining, the THP-1 cells were stimulated with 0, 5 and 10 μg/ml GST-Cpn 0810 for 24 h. Reduced nuclear size and blocky/granular particles were observed in the cytoplasm of the THP-1 cells following treatment with 5 μg/ml GST-Cpn 0810. When the concentration was increased to 10 μg/ml GST-Cpn 0810, the morphological changes were more evident (Fig. 3).

**Figure 4:**
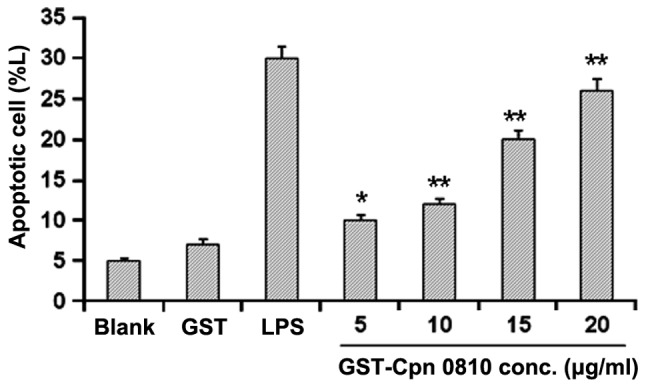
Annexin-V-FITC-PI staining of THP-1 cells treated with GST-Cpn 0810. THP-1 cells were stimulated with 1–5 μg/ml GST-Cpn 0810 or 0.1 μg/ml LPS for 24 h, stained with annexin-V-FITC-PI, and analyzed by fluorescence-activated cell sorting. The percentages of apoptotic cells were calculated and statistical analysis was performed. ^*^P<0.05 and ^**^P<0.01, vs. GST control group. FITC-PI, fluorescein isothiocyanate-propidium iodide; GST, glutathione S-transferase; LPS, lipopolysaccharide.

Analysis of annexin V binding in the THP-1 cells was performed using annexin V-FITC detection kits, following treatment with GST-Cpn 0810 for 24 h. In the flow cytometric analysis, control cells exhibited weak FITC and PI signals, early apoptotic cells showed high PI but low FITC fluorescence, and late apoptotic or necrotic cells showed strong FITC and PI staining. GST-Cpn 0810 treatment for 24 h was shown to induce apoptosis in the THP-1 cells in a dose-dependent manner, with a significantly increased apoptotic rate of 25.84±5.50% at 20 μg/ml (P<0.01). The apoptotic rate in these cells was 30.05±3.18% when treated with LPS (positive control), indicating that GST-Cpn 0810 significantly promoted apoptosis in the THP-1 cells (Fig. 4).

## Discussion

*C. pneumoniae* causes a variety of diseases in humans, including chronic infection, lung disease and cardiovascular disease; however, its pathogenic mechanism is not fully understood. Previous studies have shown that Cpn T3SS may play an important role in these pathogenic processes, and Cpn 0810 has been predicted to be one of its effector proteins. In the present study, the Cpn 0810 gene was subcloned into the prokaryotic expression vector, pGEX6p-2, and successfully transformed into *E. coli* BL21 cells. The recombinant proteins were purified using a GST purification resin column, and applied to THP-1 cells in order to study the effects.

The Cpn 0810 recombinant protein was shown to stimulate THP-1 cells to produce proinflammatory cytokines, including TNF-α and IL-6, in a dose- and time-dependent manner. The TNF-α and IL-6 levels increased as the GST-Cpn 0810 concentrations were increased from 0.5 to 4 μg/ml. However, as the Cpn 0810 concentration continued to increase, the inflammatory cytokine levels decreased. These results indicated that high concentrations of GST-Cpn 0810 can have toxic effects in THP-1 cells, as evidenced by the decreased levels of inflammatory cytokines. Since the proinflammatory cytokine levels in the GST control group were similar to the negative control group, the possibility of direct induction of these proinflammatory cytokines by GST was excluded. Cpn 0810 may interact with the host cell and participate in pathogenic processes. The expression levels of proinflammatory cytokines were stimulated 6 h after the administration of GST-Cpn 0810, and peaked at 24 h, demonstrating the time-course of the inflammation-stimulating effects of Cpn 0810.

TNF-α and IL-6 are important inflammatory mediators. TNF-α exerts a wide range of biological effects, and is one of the main cytokines involved in inflammatory cascades. TNF-α plays a key role in the regulation of inflammatory processes in atherosclerosis, mainly through the TNF-receptor 1 (p55) signaling pathway (20–24). Early in inflammation, TNF-α can promote immune cells in response to the invasion of pathogenic microorganisms. In addition, high levels of TNF-α can induce the instability of atherosclerotic plaques. Therefore, TNF-α has been regarded as an inflammatory biological marker for atherosclerotic plaque inflammation. TNF-α has been shown to be produced by local atherosclerotic plaque macrophages, blood neutrophils and monocytes, particularly in cases of arterial injury, plaque rupture and ulceration (25). TNF-α also activates endothelial and white blood cells, promotes the aggregation of inflammatory cells and promotes the release of inflammatory mediators (26).

IL-6 is an additional inflammatory cytokine with a variety of biological functions, known to be involved in immune regulation and the inflammatory response. IL-6 can increase the activation of platelets and fibrinogen, leading to increased blood viscosity and endothelial damage (27). In addition, IL-6 is closely associated with TNF-α in the acute phase inflammatory response, where TNF-α induces IL-6 and other factors to stimulate the production of C-reactive protein (CRP) in the liver (28). Furthermore, IL-6 and CRP are independent risk factors for stroke and myocardial infarction. *C. pneumoniae* may stimulate smooth muscle cells to produce IL-6, and *C. pneumoniae*-infected mononuclear cells may also secrete IL-6 in response to pathological factors.

A previous study found that the mortality rate of individuals with cardiovascular diseases in a five-year follow-up period was able to be predicted based on the elevated concentration of IL-6 in the peripheral blood (29). Arterial wall injury and repair, and atherosclerotic plaque formation are always accompanied by inflammation. It is widely accepted that inflammation is not only associated with the formation of atherosclerotic plaques, but is also involved in their stability, increasing the deterioration of acute coronary syndrome (30). Based on these studies, *C. pneumoniae* infection was hypothesized to increase the levels of TNF-α and IL-6, in which GST-Cpn 0810 may play a key role.

Apoptosis is a gene-regulated process of programmed cell death. Through detecting atherosclerotic plaques in the coronary and carotid arteries using TUNEL, previous studies (31,32) found that there were DNA fragments in the damaged intima. These fragments, which indicated apoptotic processes, were not detected in healthy blood vessels. In the present study, GST-Cpn 0810 was shown to inhibit THP-1 cell proliferation by inducing apoptosis. When treated with GST-Cpn 0810, typical morphological changes of apoptosis, including cell shrinkage, nuclear fragmentation, cell foam and apoptotic body formation, were observed in the THP-1 cells. With annexin V-FITC apoptosis detection kits, apoptotic processes were also detected at 24 h after GST-Cpn 0810 stimulation in these cells.

In conclusion, the results of the present study demonstrated that recombinant GST-Cpn 0810 induces the expression and secretion of the proinflammatory cytokines, TNF-α and IL-6, and promotes apoptotic processes in THP-1 cells. Therefore, Cpn 0810 may function as an important pathogenic factor in interactions with host cells. However, further studies are required to reveal the underlying mechanisms of these processes.
